# 全孔道人工气胸下四臂机器人肺段切除术的临床应用

**DOI:** 10.3779/j.issn.1009-3419.2022.101.52

**Published:** 2022-11-20

**Authors:** 玉龙 陈, 辉 陈, 峰 徐, 冰生 孙, 健 尤

**Affiliations:** 300060 天津，天津医科大学肿瘤医院肺部肿瘤科，肿瘤研究所，国家癌症临床研究中心，肿瘤防治重点实验室，天津市肿瘤临床研究中心 Department of Lung Cancer, Tianjin Medical University Cancer Institute and Hospital, National Clinical Research Center for Cancer, Key Laboratory of Cancer Prevention and Therapy, Tianjin's Clinical Research Center for Cancer, Tianjin Lung Cancer Center, Tianjin Cancer Hospital Airport Hospital National Clinical Research Center for Cancer, Tianjin 300060, China

**Keywords:** 肺肿瘤, 机器人辅助胸外科, 肺段切除术, 全孔道, 人工气胸, Lung neoplasms, Robot assisted thoracic surgery, Segmentectomy, Port-only, Artificial pneumothorax

## Abstract

**背景与目的:**

目前机器人手术在胸外科得到了广泛的应用，机器人手术有着更高的操控性、精确性和稳定性，尤其对于小空间复杂操作及重建手术，优势更为明显，而全孔道人工气胸下机器人行肺段切除术具有明显的操作优势。

**方法:**

基于大量的临床实践，我们建立了一整套全孔道人工气胸下四臂机器人肺段切除的手术方法。2019年1月-2022年8月，我们用此方法完成机器人肺段切除手术98例，现将临床经验进行总结。

**结果:**

全孔道人工气胸下机器人行肺段切除术在肺段血管、支气管的解剖方面有明显优势，具有出血少、手术时间短、暴露充分、操作灵活等特点。

**结论:**

我们提出的这套手术模式优化了肺段切除的操作方式及技巧，使每一个步骤程序化，减轻副损伤，且易于学习掌握，相信能够以更小的创伤治愈更多的肺癌患者。

## Abstract (en)

**Background and objective:**

At present, robotic surgery is widely used in thoracic surgery, which has higher maneuverability, precision, and stability, especially for small space complex operations and reconstructive surgery. The advantages of robotic lung segment resection under full orifice artificial pneumothorax are obvious.

**Methods:**

Based on a large number of clinical practices, we established a set of surgical methods for 4-arm robotic lung segment resection under a port-only artificial pneumothorax. 98 cases of robotic lung segment resection were performed with this method from January 2019 to August 2022. The clinical experience was summarized.

**Results:**

Robotic lung segment resection under port-only artificial pneumothorax has obvious advantages in the anatomy of lung segment vessels and bronchi. It is characterized by less bleeding, shorter operation time, adequate exposure, and flexible operation.

**Conclusion:**

This surgical model we propose optimizes the operation mode and technique of lung segment resection, makes each step procedural, reduces collateral damage, and is easy to learn and master, which is believed to cure more lung cancer patients with less trauma.

机器人手术作为一种外科新技术，是微创手术领域的重要进步^[1]^。在泌尿外科、普通外科、胸外科、心脏外科和头颈外科手术中，这种创新的技术已经得到了广泛的应用，成为了外科手术的重要支柱。达芬奇机器人手术系统（Da Vinci surgical robot）作为一种高级外科机器人平台，旨在通过微创的方法实施复杂的外科手术。主刀可在手术无菌区外控制台或远程进行手术操作，安装在一个手臂上的双摄像头内窥镜将手术区域的图像传输到控制台，为外科医生提供放大的三维视图。而手术助手则辅助操作无菌区内的床旁机械臂系统，负责更换器械及应用切割闭合器等，协助主刀医师完成手术。随着机器人系统不断升级，助手的作用不断弱化。尽管胸腔天然的骨性结构使机器人打孔位置有所限制，但天然的操作空间也使机器人手术在胸外科得到了广泛的应用。机器人手术的天然优势就是狭小空间的精细操作，肺段切除术正是契合了这种方式^[2]^。在本研究中，我们总结了我科应用全孔道人工气胸下机器人肺段切除术行肺段切除的病例，并对该手术方案的优势进行讨论，以期为临床策略的制定提供依据。

## 1 资料与方法

### 1.1 一般资料

在总结发展我们以前报道的全孔道人工气胸下四臂技术肺叶切除术经验的基础上，2019年1月-2022年8月我们用此方法完成机器人肺段切除术98例。具体资料汇总：98例中，男27例，女71例；年龄30岁-74岁，平均年龄57.5岁。病变位于右肺S1段5例，右肺S2段9例，右肺S3段8例，右肺S6段8例，右肺S8段4例，右肺S10段1例，右肺S9+10段4例，左肺S1+2段25例，左肺S3段5例，左肺S4+5段5例，左肺S6段10例，左肺S8段2例，左肺S10段1例，左肺星段（*段）1例，左肺S6+10段2例，左肺S1+2+3段3例，联合亚段、亚段切除8例。其中联合肺段切除7例。适应证与常规胸腔镜手术相同，在综合考量肿瘤胸膜侵犯、生长方式、病理亚型等方面因素后，对具有毛玻璃成分的临床I期可手术患者行机器人辅助解剖性肺段切除术。

### 1.2 体位和麻醉

全身麻醉，双腔插管，90°侧卧折刀位，胸下垫塑形垫固定体位，不用托手架，患侧腋下垫软垫，不影响机械臂活动。适当折刀位避免镜头机械臂对髋骨的压迫，增大肋间隙（图 1）。手术野常规消毒铺巾，术前打孔采用常规罗哌卡因肋间及椎旁阻滞，减少穿刺器对肋间神经的压迫损伤，减轻术后伤口疼痛。

**图 1:**
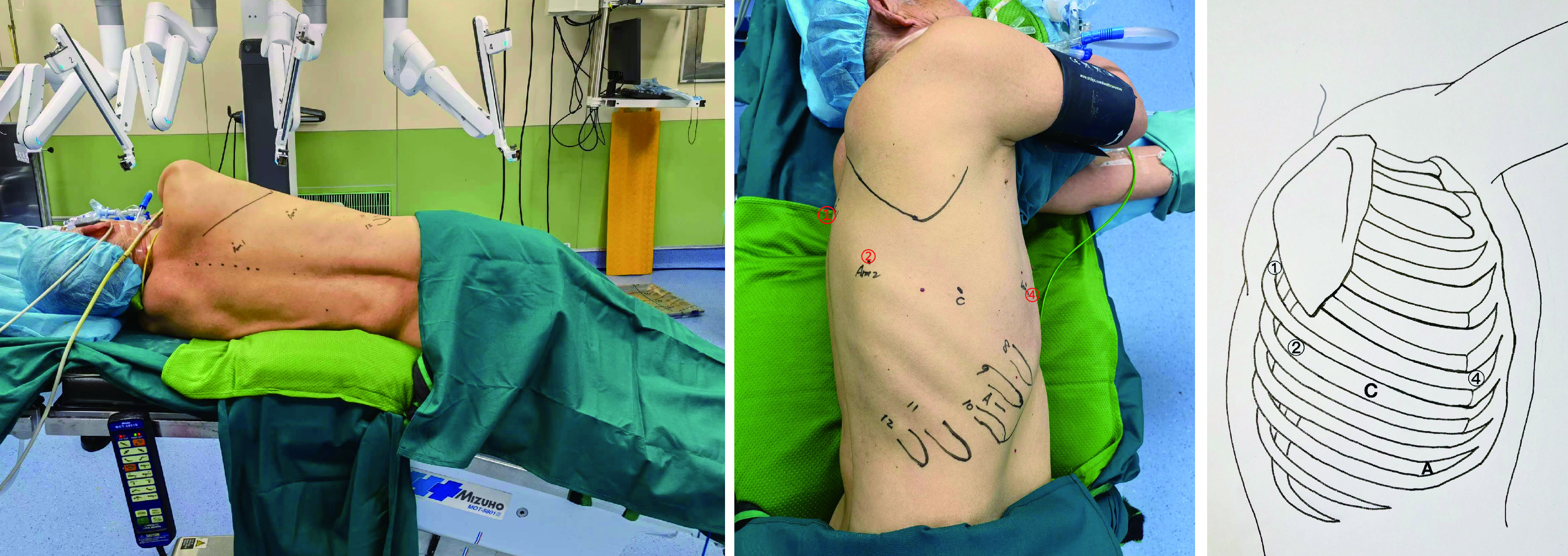
患者体位及打孔位置。左图：患者体位：侧卧位轻度弯曲以扩大肋间隙；中图和右图：打孔位置。C：镜头孔；A：辅助操作孔。①: 背部操作孔；②: 后部操作孔；④: 前部操作孔。 Port placement and patient positioning. Left figure: patient positioning. Patient's position is lateral decubitus on bended table to expand intercostal space; Middle and right figures: Port placement. C: camera port; A: assistant port; ①: back port; ②: posterior port; ④: anterior port.

### 1.3 孔道布局

机器人系统为DaVinci Xi system（Intuitive Surgical, Sunnyvale, CA）。所有操作孔道建立均可视化，电刀仅切开皮肤及皮下组织，并不切开肌层，而由穿刺器钝性分离进入胸腔。首先建立镜孔（8 mm）选在腋中线第7或8肋间，机器人镜头30°向上，检查胸腔有无广泛粘连，于镜孔建立人工气胸，压力4 mmHg-6 mmHg，流量12 L/min-15 L/min；然后可视下建立辅助操作孔（12 mm），通常位于第9肋间，主要协助压肺、放置带开关吸引器和切割缝合器以及拿取标本及淋巴结；机器人镜头和气胸管移至辅助操作孔，在全可视下建立后部操作孔（8 mm）和前部操作孔（8 mm），根据患者体型及左右肺不同可选腋前线稍微偏前和肩胛下角线稍偏后与镜孔同一肋间或上下移1个肋间，原则上确保后部的操作孔位置低于斜裂；背部操作孔（8 mm）位于脊柱旁线、第5肋间，根据不同胸廓形状调整（图 1）。常用器械包括8 mm马里兰钳（Maryland Bipolar Forceps）、8 mm无损伤抓钳（Cardiere Forceps）、8 mm（Tip-Up Fenestrated Grasper）及针持等，并且应用自制小纱布卷推压肺组织暴露解剖部位，极大地减少对正常肺组织的牵拉钳夹。术毕，适当延长辅助操作孔取标本，因为辅助孔位于肋弓膈肌反折处，组织间隙较大，易于取出标本。所有肺段的打孔位置和方式基本恒定（因左右肺各机械臂对应的数字不一样，故文中统一以前部，后部，背部操作孔命名）。打孔流程视频见http://www.lungca.org/files/video1.mp4。

### 1.4 手术方式及流程优化

#### 1.4.1 右肺上叶诸段

##### 1.4.1.1 右肺上叶S1段

背部操作孔Tip-Up夹持纱布卷向后下方推开上肺，暴露上肺静脉和上部肺门，解剖上肺静脉，向远端游离出第一属支之V^1^a、V^1^b分叉处，心包抓钳和马里兰钳配合结扎切断V^1^a，保留V^1^b系与S3段分界的段间静脉；进一步解剖上部肺门，暴露肺动脉之A^1^，如存在A^2^返支，注意保留，同样方法结扎或切割缝合器切断A^1^，解剖尖段支气管后切割缝合器切断。静脉快速注射吲哚菁绿后，切换Firefly模式，马里兰钳标记尖段的脏层胸膜表面；提起段门支气管断端，沿段间平面适当游离段门。最后切割缝合器沿标记线完整切除右肺上叶尖段（文中所有切割缝合器均由低位辅助孔进入，不再赘述）。

##### 1.4.1.2 右肺上叶S2段

背部操作孔Tip-Up夹持纱布卷向头侧推开上肺，暴露叶裂后部，解剖暴露叶裂处肺动脉下干和后升支动脉；Tip-Up向前推开上叶，解剖上叶支气管至暴露前段支气管；切割缝合器分离后部斜裂后，辨认后段支气管和后升支动脉，以切割缝合器和结扎切断；Tip-Up提起后段支气管断端，马里兰钳游离段门，解剖中心静脉属支V^2^a、V^2^b、V^2^c、V^3^a，结扎切断V^2^b，注意保留V^3^a和V^2^a；同样吲哚菁绿标记后段范围后，切割缝合器沿标记线完整切除右肺上叶后段。

##### 1.4.1.3 右肺上叶S3段

背部操作孔Tip-Up夹持纱布卷向头侧推开上肺，暴露水平裂，解剖暴露叶裂处肺动脉下干和中心静脉，Tip-Up向后方推开上叶，暴露上肺静脉，解剖V^1^和中心静脉各属支；切割缝合器分离发育不全水平裂；结扎或者锁扣夹切断V^3^b、V^3^a；Tip-Up向脚侧推开上叶，暴露上部肺门，解剖尖前动脉干暴露A^1^、A^3^；Tip-Up向后部推开上叶，适当牵开A^1^，结扎或锁扣夹处理A^3^动脉，解剖前段支气管，切割缝合器切断，注意：B^3^a亚段支气管深在容易遗漏，仔细辨认支气管切除平面，并且注意保护深面的中心静脉；提起前段支气管断端，马里兰钳沿段间静脉游离段门。吲哚菁绿标记前段范围后，切割缝合器沿标记线完整切除右肺上叶前段。右上肺S3段视频见http://www.lungca.org/files/video3.mp4。

#### 1.4.2 左肺上叶诸段

##### 1.4.2.1 左肺上叶S1+2+3段（固有段）

背部操作孔Tip-Up夹持纱布卷向头侧推开上肺，暴露叶裂后部，解剖暴露叶裂处肺动脉下干，舌段动脉和A^1+2^c作为标志；Tip-Up向前推开上叶，暴露后部肺门，解剖出肺动脉总干和上叶后部的A^1+2^c和A^1+2^a+b分支；切割缝合器分离后部斜裂；Tip-Up向脚侧推开上叶，暴露主动脉弓下肺门，解剖肺动脉干和上叶分支，辨认A^3^、A^1+2^c、A^1+2^a+b，分别以切割缝合器或者结扎切断，如存在纵隔型舌段动脉，注意辨认保护。在舌段动脉后侧解剖固有上叶支气管，注意暴露出舌段支气管，以切割缝合器切断。Tip-Up提起固有段支气管断端，马里兰钳游离段门前部静脉属支V^1+2^、V^3^c、V^3^a+V^1+2^d，分别结扎切断，注意保留与舌段段间静脉V^3^b；吲哚菁绿标记S1+2+3段范围后，切割缝合器沿标记线完整切除左肺上叶S1+2+3段（第一次击发建议由前段和舌段交界开始）。

##### 1.4.2.2 左肺上叶S1+2段

解剖步骤基本同左上固有段切除，动脉分支只需要切断A^1+2^c、A^1+2^a+b分支，在舌段动脉偏后侧解剖固有上叶支气管，注意暴露出舌段支气管和B^3^支气管，注意保护B^1+2^前方的V^1+2^d段间静脉，切割缝合器切断B^1+2^后，Tip-Up提起支气管断端，马里兰钳游离段门前部静脉属支V^1+2^b, c，分别结扎切断。同样吲哚菁绿标记S1+2段范围后，切割缝合器沿标记线完整切除左肺上叶S1+2段。

##### 1.4.2.3 左肺上叶S3段

背部操作孔Tip-Up夹持纱布卷向后下方推开上肺，暴露弓下和前上部肺门，分离肺动脉和上肺静脉交界，解剖肺动脉总干，显露A^3^分支，调整Tip-Up向后方拉开上肺，显露上肺静脉，解剖出V^3^c，予以结扎或者锁扣夹切断，保留V^3^b，并向远端解剖；适当游离V^1+2^a-c后，分离其后方之A^3^，以切割缝合器或结扎切断，调整Tip-Up位置，推开切断之血管断端，暴露出固有段支气管，适当向远端游离，辨认B^3^和后方之B^1+2^及下方的舌段支气管，以切割缝合器切断；Tip-Up提起前段支气管断端，马里兰钳沿段间静脉V^3^b、V^1+2^d和V^1+2^a-c游离段门；吲哚菁绿标记前段范围后，切割缝合器沿标记线完整切除左肺上叶前段。

##### 1.4.2.4 左肺上叶S4+5段（舌段）

背部操作孔Tip-Up夹持纱布卷向上方推开上肺，叶裂中部最薄弱处解剖叶间肺动脉，暴露A^4+5^和A^8^分叉；调整Tip-Up向后方推开上肺，解剖上肺静脉，游离结扎切断V^4+5^，暴露后方舌段支气管和下叶支气管分叉；切割缝合器结合锁扣夹分离发育不全之斜裂前部；充分游离A^4+5^后结扎切断；游离舌段支气管，以切割缝合器切断。Tip-Up提起舌段支气管，马里兰钳向远端游离段门，吲哚菁绿标记舌段范围后，切割缝合器沿标记线完整切除左肺上叶舌段。

#### 1.4.3 下叶诸段

##### 1.4.3.1 下叶S6段

背部操作孔Tip-Up夹持纱布卷向前上方推开下肺，游离下肺韧带，解剖下肺静脉，显露并充分游离V^6^属支，注意充分分离与支气管粘连；Tip-Up向上推开上叶，暴露叶裂后部，解剖出A^6^，切割缝合器分离斜裂后部，切断A^6^，充分游离B^6^后，切割缝合器切断，牵拉B^6^支气管断端，游离V^6^a切断，注意保留V^6^b, c段间静脉；进一步提起支气管断端后，沿段间静脉向远端游离段门，吲哚菁绿标记背段范围后，切割缝合器沿标记线完整切除背段。

##### 1.4.3.2 下叶S8段

背部操作孔Tip-Up夹持纱布卷向后上方推开下肺，游离下肺韧带，解剖下肺静脉，暴露下肺静脉的前部上下基底静脉，适当向远侧游离；Tip-Up向上推开舌叶或者中叶，解剖叶裂处叶间动脉干，游离A^8^，多数单独发出，切割缝合器或者结扎切断；如有发育不全斜裂，以切割缝合器切开；Tip-Up或者助手牵开A^8^断端，解剖下方的B^8^支气管，闭合切断；提起B8残端，辨认同向走形的V^8^a，结扎切断，注意尽量保留V^8^b系与S^9^的段间静脉，沿V^8^b向远端游离段门后，吲哚菁绿标记S^8^段范围后，切割缝合器沿标记线完整切除前基底段（注意第一枪由膈面靶段界限开始分离）。右下肺S8段视频见http://www.lungca.org/files/video2.mp4。

##### 1.4.3.3 下叶S10段

背部操作孔Tip-Up夹持纱布卷向上方推开上肺，叶裂后部解剖叶间动脉干，解剖出A^6^和A^10^动脉，辨认后结扎切断；Tip-Up向前上方推开下肺，游离下肺韧带，充分解剖下肺静脉V^6^、上、下基底静脉三属支，V^10^静脉多位于下基底静脉最下方一支，结扎切断；Tip-Up和助手协助牵开上下基底静脉，解剖B^10^支气管，切割缝合器切断；Tip-Up提起B^10^断端，向远端游离段门，吲哚菁绿标记S10范围，切割缝合器沿标记线完整切除后基底段（注意由膈面靶段界限开始分离）。S9段多与S10段共干，S9+10段切除步骤和上述S10段基本相同，不再赘述。

##### 1.4.3.4 星（*）段

背部操作孔Tip-Up夹持纱布卷向上方推开上肺，叶裂处解剖叶间动脉干，解剖出星段动脉，有时星段动脉分出二支，结扎切断。下方就是星段支气管，以切割缝合器切断。Tip-Up提起支气管断端，向远端游离段门，吲哚菁绿标记星段范围，切割缝合器沿标记线完整切除。

#### 1.4.4 淋巴结清扫

肺段切除多应用于磨玻璃结节，纵隔淋巴结清扫多数只是采样。机器人下清扫较常规胸腔镜更为容易，马里兰钳配合Cardiere钳，Tip-Up抓持小纱布卷推拉组织暴露，几乎不需吸引器的暴露及吸引，无论对于隆突下淋巴结还是上纵隔淋巴结均易于切除，而且由于马里兰钳双极电凝止血彻底，产烟极少，能充分保证术野干净清晰。

### 1.5 手术操作技巧

#### 1.5.1 马里兰钳操作技巧

利用马里兰钳的双极电凝，可以极其安全地在大血管周围解剖，不必担心周围副损伤。马里兰钳也可以配合心包抓钳结扎血管后切断，也可以简单缝合组织，甚至可以离断缝线。

#### 1.5.2 血管处理技巧

多数血管可以马里兰钳分离后，通过低位辅助孔切割缝合器切断。较细或者进切割缝合器角度不顺畅的血管，可以马里兰钳和Cardiere钳配合结扎后切断，内镜下的锁扣夹也是可以选择的处理方式。

#### 1.5.3 支气管处理技巧

肺段支气管位于肺实质内，而且周围多伴淋巴结粘连干扰，对于伴陈旧炎性病变较重的患者，充分解剖靶段支气管相对较困难，可以应用马里兰钳直接切断靶段支气管，并充分游离后，以切割缝合器二次闭合。这种技巧可以使粘连的支气管易于正确切断，而且使切割缝合器施放角度更加轻松。

#### 1.5.4 肺裂发育差的处理技巧

某些肺段如右肺上叶S1段和左肺上叶S1+2段的a+b亚段，采用由上往下的方式解剖血管和气管，可以采用不打肺裂的手术方式。而下叶的基底段，如S7、S8、S9、S10段，如果静脉不是很复杂，也可以采用由下往上的解剖顺序，先解剖离断肺段的静脉，然后解剖肺段气管及动脉，这样的优点是损伤较小，缺点是静脉、动脉和气管辨认有难度，需要非常熟练掌握后才可以做到。其他的肺段，需要先以马里兰钳以及腔内一次性切割缝合器打开肺裂。技巧是从斜裂的中间部位、动脉最表浅的地方开始，找到肺动脉的层次，然后解剖后纵隔胸膜，马里兰钳可以很容易打通肺裂的隧道，然后以腔内一次性切割缝合器打开肺裂。

#### 1.5.5 三维重建

因机器人手术无触觉和力反馈，且全孔道操作，应该减少翻动肺脏，避免戳卡在肋间过多大范围转动引起孔道出血，故建议常规三维重建靶段结构，利于术中辨认和正确处理。

## 2 结果

全组无围术期死亡。1例因粘连严重，肺动脉干出血中转开胸。全组手术时间为35 min-170 min，平均为86.5 min。前50例平均手术时间为98.2 min，后48例手术时间为74.3 min。术中出血量为5 mL-500 mL。其中前50例术中中位出血量为20 mL（5 mL-500 mL），后48例为20 mL（5 mL-60 mL）。所有手术患者无围手术期严重手术并发症（支气管胸膜瘘、肺栓塞、术后胸腔出血、乳糜胸、严重肺感染等）或术后死亡情况（表 1）。

**表 1:** 全孔道人工气胸下肺段切除术手术相关和术后结果 Surgery-related and postoperative outcomes of port-only robot-assisted segmentectomy

Items	First 50 cases	Subsequent 48 cases
Port employment & docking time (min)	17.3±7.2	9.1±3.8
Operative time (min)	98.2±39.0	74.3±31.8
Bleeding volume (mL)	20 (5-500)	20 (5-60)
Serious complications*	0	0
Postoperative morbidity	0	0
Hospital stay (d)	3.6	2.4
Serious complications*: Bronchopleural fistula, pulmonary embolism, postoperative thoracic hemorrhage, chylothorax, severe pulmonary infection, *etc*.		

## 3 讨论

近年来，随着临床诊断学技术的提高，如低剂量螺旋计算机断层扫描（computed tomography, CT）的筛查，高分辨率CT三维成像等用于诊断肺部微小病灶，以及胸部CT逐渐纳入常规的体检项目，肺磨玻璃病变及肺小结节的检出率逐年增高^[3]^。肺磨玻璃病变的手术治疗逐渐成为目前胸外科手术的重点^[4]^。鉴于肺叶切除损伤范围较为广泛，对肺功能的影响相对较大，而大部分肺磨玻璃结节发生转移的几率极低。因此，在对磨玻璃结节的大小以及浸润程度综合评估后，肺段或亚段切除术逐渐成为目前大部分肺磨玻璃结节手术治疗的主流术式^[5]^。两项多中心前瞻性临床试验（JCOG0802/WJOG4607L和CALGB/ALLIANCE 140503）结果^[6, 7]^表明，肺叶切除术和节段切除术在功能正常患者的围手术期死亡率和发病率方面几乎没有差异。JCOG0802/WJOG4607L试验^[8]^报道了其随访结果，揭示了节段切除术可能比肺叶切除术更能提供生存和功能上的益处。这进一步奠定了肺段切除术在肺磨玻璃病变手术治疗中的地位。

机器人手术的天然优势就是狭小空间的精细操作；而肺段血管和气管多较细小，需要特别精细的操作，故应用机器人行肺段切除手术具有天然的优势。我们建立的手术方式与其他机器人手术的区别：首先所有操作孔道建立均可视化。先建立镜孔，检查胸腔有无广泛粘连，于镜孔建立人工气胸，然后可视下建立辅助操作孔，一般选在肋弓膈肌反折处，这里的组织间隙较大，易于取出标本，更直观，创伤也最小。其余各臂的孔道建立也是在可视下完成，这样保证了打孔的精确性。其次，我们建立的方法应用了四臂技术，四臂技术的应用，使术者在术中可以自行暴露术野，减轻肺组织牵拉损伤，减少对助手的依赖，保证手术质量的恒定；同时随着四臂操作技术熟练程度的提高，手术时间也会缩短，使手术创伤更轻^[9, 10]^。

另外，我们的方法采用的是应用人工气胸的全孔机器人手术（robotic port-based lobectomy, RPL），与机器人辅助胸腔镜手术（robotic access-incision lobectomy, RAL）相比，RPL技术是在胸腔完全封闭的情况下进行的，通过注入温暖二氧化碳气体使胸廓膨胀，肺组织更好地萎陷，膈肌下移，提供了充分的操作空间，尤其对于肥胖或左室肥大的左肺手术患者改善术野显露的效果更加明显；另外低压力人工气胸可以扩大纵隔间隙，使解剖层次清晰，减少细微血管的渗出，使术野干净；此外还避免了柔软的肺组织直接与手术室干燥寒冷的空气接触^[11-13]^。多项对照研究^[13, 14]^显示，RPL的术中失血相较RAL更少，可能是暴露清晰，胸腔内压力的增加抑制了组织的渗血。同时，RPL的手术时间较RAL更短，可能是由于RPL策略中术者亲自暴露术野，减少了对助手的依赖；另外RPL手术空间大，手术过程平稳，术者体验更好，从而减轻了手术的压力^[15]^。

手术中我们主要应用的能量器械是马里兰双极钳，马里兰钳的尖端比较纤细，相比电钩来说，更容易解剖肺段切除操作中较细血管和气管周围的组织，不容易损伤到很细的段和亚段的血管和气管，这方面对于肺段切除尤为重要。马里兰钳不仅仅用于解剖，还可以和Cardiere钳配合结扎打结甚至简单的缝合，还可以切断血管，气管甚至缝线，术中几乎无需更换其他器械，极大地减少了手术费用和时间。由于肺段支气管位于肺实质内，而且周围多伴淋巴结粘连，伴陈旧炎性病变的患者尤为严重，充分解剖靶段支气管相对较困难。建议的技巧是：可以应用马里兰钳直接切断靶段支气管，并充分游离后，以切割缝合器二次闭合。这种方式可以使粘连的支气管易于辨识正确切断，而且使切割缝合器施放角度更加轻松。另外对于各不同肺段的解剖顺序，我们也根据自己的经验制定了不同的策略，这些优化的操作步骤，可以使每个肺段操作更加容易和便捷，也更安全。

人工气胸下全孔道机器人手术的不足之处：如果有较大的血管出血，需要持续吸引，会引起压力不足，而导致肺被动膨胀；这可以通过提前暴露好操作视野、术中细致操作，以减少术中出血的发生；同时也可以通过恒压气腹机解决。如果应用电钩的话，烟雾的量较大，偶尔会需要中断操作等待烟雾排出。

无疑机器人的机械臂具有超越人手的灵活性、稳定性，可以非常精细地进行肺段切除手术。我们提出的这套手术模式，优化了肺段切除的操作方式及技巧，使每一个步骤程序化，减少副损伤，且易于学习掌握，此技术的广泛开展，相信能够以更小的创伤治愈更多的肺癌患者。
